# Virchow and the Darwinian Theory

**Published:** 1890-02

**Authors:** 


### Virchow and the Darwinian Theory.

According to the Vienna correspondent of the *British Medical Journal*, in Professor Virchow's presidential address at the recent meeting of the Anthropological Congress, the Darwinian theory was referred to, and he said that the intermediate link that should bring man and the ape into connection—the proper "prosanthropos"—had been sought for in vain. It was impossible even to determine the descent of single races from others; and it could be asserted that among the ancient races there was none that stood in any nearer relationship to the ape than ourselves. There was no tribe of people in the world that we were unacquainted with, and not one of the known tribes could justly be considered ape-like, appearances common to apes—such as prominences of the skull—being insufficient evidence of relationship. There was evidence that in the course of 5,000 years no remarkable changes of type had taken place. This adds to the evidence of the impassibility of the chasm between the highest type of anthropoid ape and lowest type of man.—*N. Y. Med. Journal*

